# Pitfalls of laparoscopic Re-TAPP in recurrent inguinal hernia repair—a plea for extended preoperative diagnostic

**DOI:** 10.1093/jscr/rjab085

**Published:** 2021-03-29

**Authors:** Ivana Raguz, Reint Burger, Rene Vonlanthen, Marco Bueter, Andreas Thalheimer

**Affiliations:** Department of Surgery, Spital Männedorf, Männedorf, Switzerland; Department of Surgery, Spital Männedorf, Männedorf, Switzerland; Department of Visceral and Transplant Surgery, University Hospital of Zürich, Zürich, Switzerland; Department of Surgery, Spital Männedorf, Männedorf, Switzerland; Department of Visceral and Transplant Surgery, University Hospital of Zürich, Zürich, Switzerland; Department of Surgery, Spital Männedorf, Männedorf, Switzerland; Department of Visceral and Transplant Surgery, University Hospital of Zürich, Zürich, Switzerland

## Abstract

According to international guidelines, recurrent inguinal hernia should be treated by a surgical approach opposing of the primary strategy (anterior–posterior or posterior–anterior). However, recent evidence demonstrates feasibility and safety of re-laparoscopic repair of recurrent inguinal hernia after primary laparoscopy. For such a strategy, correct identification of anatomical structures is challenging, but absolutely crucial for a satisfactory postoperative result. This case of an unrecognized sliding hernia of the sigmoid colon during re-laparoscopy highlights that a precise physical examination as well as an extended preoperative radiological workup (ultrasound, computed tomography and/or magnetic resonance imaging of the abdomen and pelvis) should be considered prior to re-laparoscopy of recurrent inguinal hernia.

## INTRODUCTION

Nearly 20 million primary inguinal hernia operations are performed globally and annually. A recurrence rates of up to 12% have been reported with <50% being reoperated. Diagnosis of a recurrent hernia can be difficult, especially when clinical symptoms are equivocal. Physical examination combined with sonography represents the standard approach. In unclear cases, magnetic resonance imaging (MRI) or computed tomography (CT) may be performed additionally [1].

The European Hernia Society and HerniaSurge Group guidelines recommend a change of the surgical strategy for reoperations, i.e. the use of posterior repair in case of primary anterior repair and vice versa [1, 2]. However, the re-laparoscopic approach of recurrent inguinal hernia after primary posterior repair gains acceptance [3–5]. Advantages of the re-laparoscopic approach include less postoperative pain, better cosmetic results and an earlier return to work and daily activities.

Herein, we demonstrate potential pitfalls of re-laparoscopic repair of recurrent inguinal hernia and highlight advantages of an extended preoperative radiological workup.

## CASE PRESENTATION

A male patient (51 years) underwent bilateral transabdominal preperitoneal (TAPP) repair for a large left-sided sliding hernia (sigmoid colon) and a small right-sided medial inguinal hernia 7 months ago. A large symptomatic recurrence on the left side was clinically confirmed.

**Figure 1:**
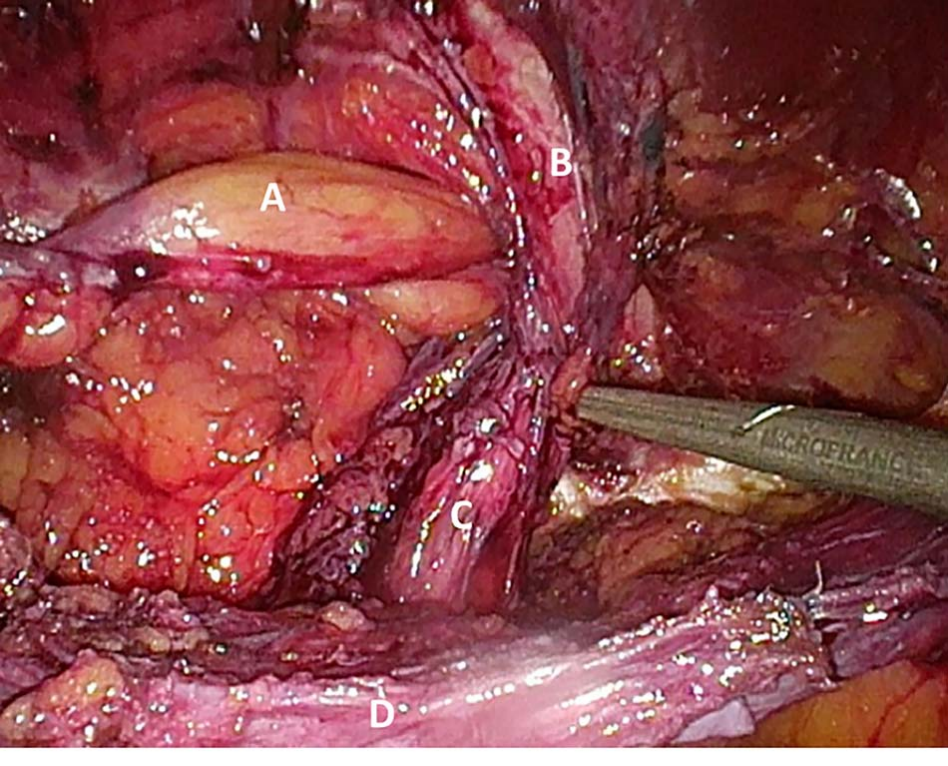
Suspected indirect inguinal recurrence hernia with a large spermatic cord lipoma (**A**), epigastric vessels (**B**), ductus deferens (**C**), peritoneum with previous mesh (**D**).

**Figure 2:**
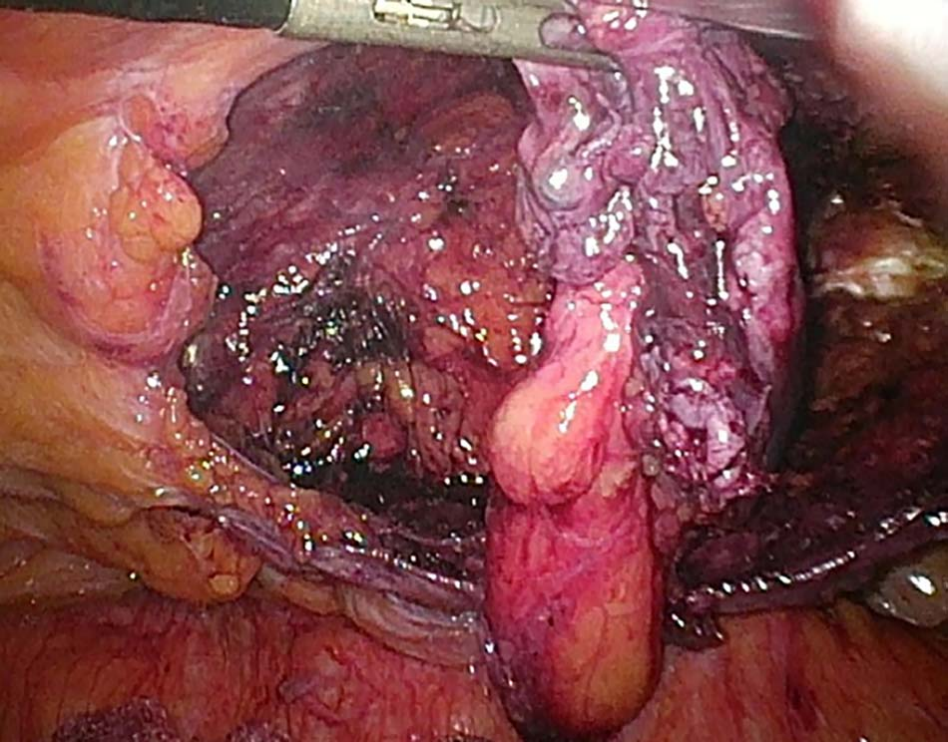
Dissection of the large spermatic cord lipoma with attached fibrotic tissue.

Considering the patients’ comorbidities (obesity, Type 2 diabetes mellitus), a re-TAPP was performed to avoid possible wound complications. The recurrent hernia was presumably confirmed as a lateral inguinal hernia. After dissecting dense fibrotic scar tissue around the previous mesh preperitoneally, the hernia sac was released. A large spermatic cord lipoma was found and dissected (Figs 1 and 2). A new mesh (BARD® 3D Light Mesh, 10 × 15 cm) was inserted.

**Figure 3:**
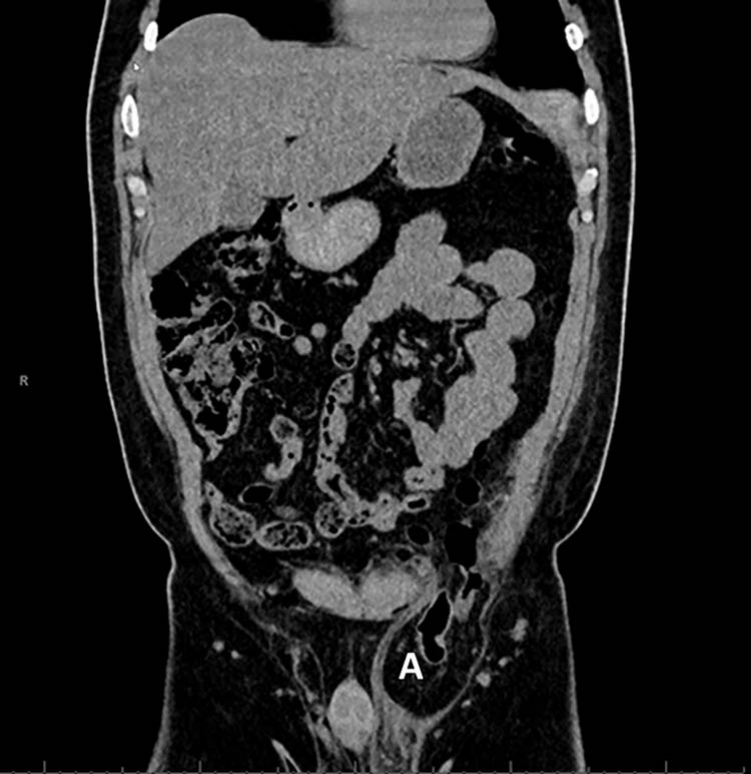
Computed tomography with sigmoid colon and solid tissue in hernia sac (**A**).

However, the patient experienced persistent swelling in the left groin. To confirm the early re-recurrence hernia, CT of the abdomen revealed a left sliding hernia with sigmoid colon (Fig. 3).

**Figure 4:**
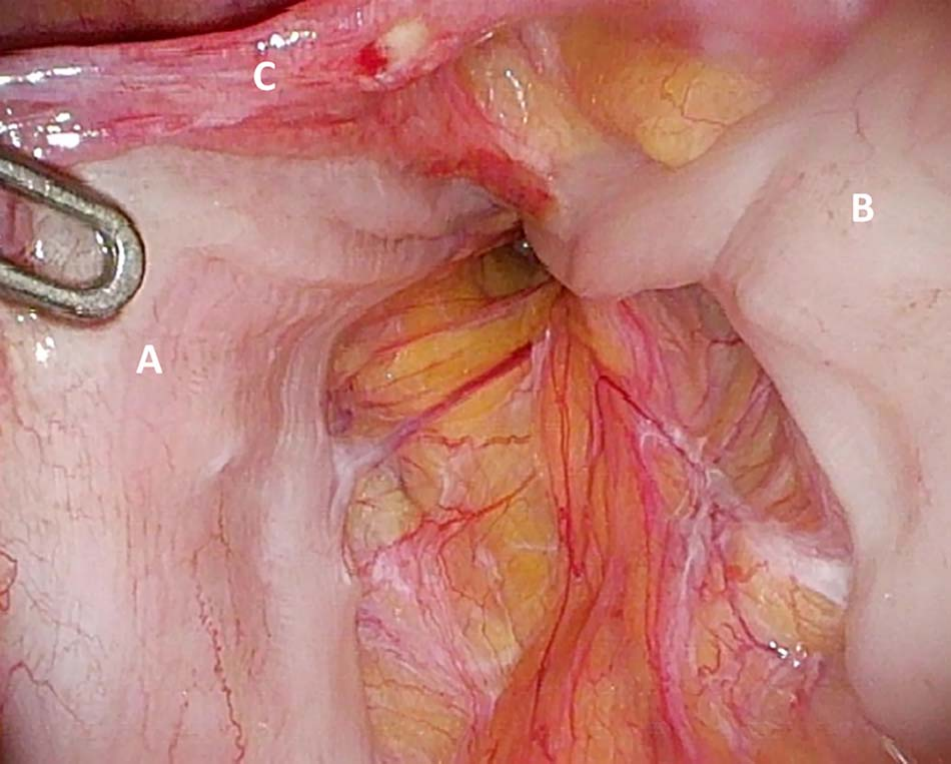
‘Loop’ of sigmoid colon in left inguinal canal (afferent loop: **A**; efferent loop: **B**; peritoneum: **C**).

**Figure 5:**
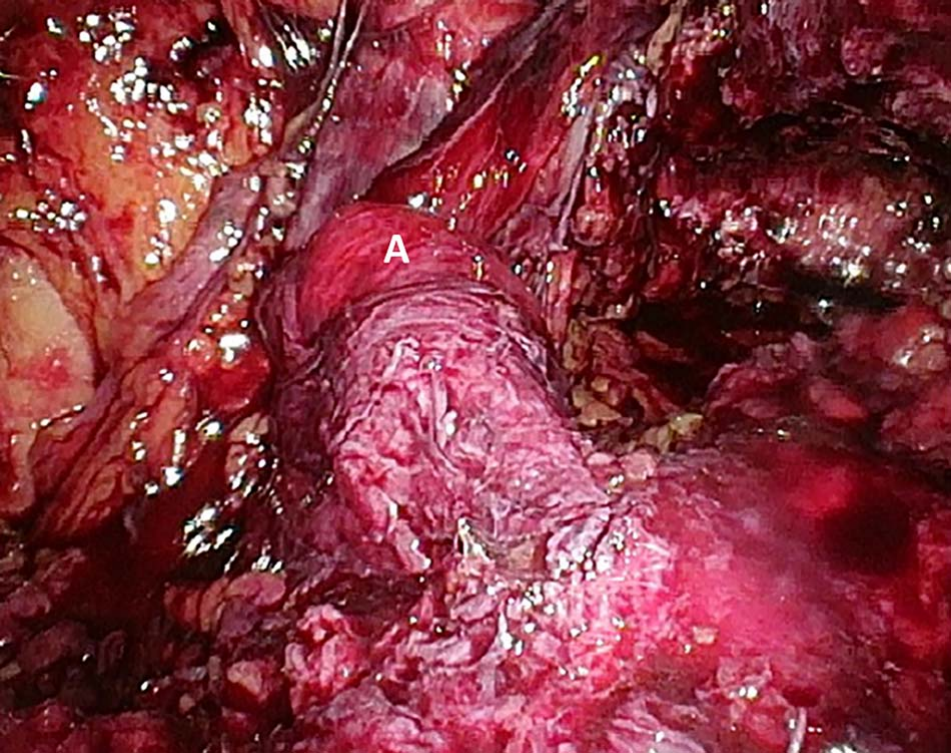
Preperitoneal view of the large hernia sac (**A**) ‘hidden’ in the scar tissue.

The patient agreed to proceed with diagnostic laparoscopy for a re-re-repair of the left inguinal hernia. Intraoperatively, a loop of the sigmoid colon, which previously was hidden by colonic adhesions, was fixed into a hernia orifice lateral–caudal of the preperitoneal mesh (Fig. 4). After reopening of the peritoneum and removal of the mesh, extensive preperitoneal preparation revealed the hernia orifice located in dense scar tissue not being dissected previously (Fig. 5). A new mesh (BARD® 3D Light Mesh 12 × 17 cm) was inserted and fixed medially at the Cooper’s ligament.

The perioperative course was uneventful with discharge at day 3. No recurrence was seen 6 months postoperatively.

## DISCUSSION

Surgery of a symptomatic inguinal hernia is one of the most often performed procedures worldwide. The primary laparo-endoscopic approach (TAPP or totally extraperitoneal (TEP)) is considered the standard procedure by many and has replaced the primary anterior technique with or without mesh (Lichtenstein/transinguinal preperitoneal technique (TIPP) or Shouldice). Although laparo-endoscopic techniques have decreased the recurrence rates significantly [6], rates of up to 12% have been reported and reoperation is often necessary [7].

Before reoperation of a recurrent inguinal hernia, physical examination and an ultrasound of the inguinal region are recommended. In addition, MRI or CT may be added in unclear cases.

There is a paucity of studies investigating the extent of preoperative radiological imaging in cases of recurrent inguinal hernia and data to support an extensive workup are scarce. A recent retrospective analysis showed 97% positive prediction of diagnosing recurrent inguinal hernia by physical examination only, suggesting that an additional ultrasound may not always be necessary [8]. As the diagnostic quality of an ultrasound is characterized by a high inter-examiner variance, its diagnostic value must even be questioned more.

The literature to support diagnosis of primary and recurrent inguinal hernia remains equivocal. Low-dose CT of the inguinal region has gained importance. Advantages include evaluation of hernia content, simultaneous investigation of the contralateral side and differentiation of other causes of inguinal swelling. In this case, a preoperative CT would have shown the sliding hernia with sigmoid colon and a sufficient surgical dissection during the first re-laparoscopy may have been performed, omitting re-re-laparoscopy.

Surgery for recurrent inguinal hernia can be challenging. An open anterior approach for the treatment of a recurrent inguinal hernia originally repaired by a laparoscopic technique, and vice versa, is recommended by international guidelines [9]. Nonetheless, successful treatment of recurrent hernias by a laparoscopic approach after primary laparoscopic surgery has also been reported [4]. Minimal invasive re-laparoscopy of primarily laparoscopic treated inguinal hernia is plausible due to its benefits: better postoperative pain control, a quicker return to activity and less postoperative wound complications.

Our patient suffered from obesity World Health Organization grade II in combination with metabolic comorbidities. Thus, it was reasonable to expect him to benefit from minimal invasive approach. After the dissection of the scar tissue, a large spermatic cord lipoma was detected and mistakenly identified as cause of hernia recurrence. No further dissection of the preperitoneal space was undertaken. This is not in accordance with recently published literature advising a dissection of the preperitoneal layer to at least 2 cm below the pubic symphysis [10]. This recommendation also applies to revisional surgery.

The re-re-laparoscopic operation confirmed a sliding hernia of the sigmoid. A complete dissection of the preperitoneal tissue was necessary. A new mesh covering the indirect, direct and femoral triangle with overlapping of the pubic symphysis was placed.

Alternative may have been a laparoscopic closure of the hernia gap, insertion of a mesh patch and preservation of the former mesh as recently proposed [11].

Although, there is no data available, robotic approach in inguinal hernia surgery may represent a beneficial alternative as it is characterized by enhanced visibility, sole control by surgeon and improved dexterity, which may refine tissue preparation even in technically demanding situations [12].

In summary, we present a case of a missed sliding hernia of the sigmoid during re-laparoscopy of a recurrent inguinal hernia following primary TAPP. We recommend extended radiological imaging including CT or MRI prior to revisional surgery. Re-laparoscopy is feasible and associated with all benefits of minimal invasive surgery. A thorough tissue preparation needs to be applied to recognize important anatomical landmarks. Robotic inguinal hernia surgery may have an important role in revisional surgery.

## FUNDING

None declared.
